# Hand Dominance and Age Have Interactive Effects on Motor Cortical Representations

**DOI:** 10.1371/journal.pone.0045443

**Published:** 2012-09-25

**Authors:** Jessica A. Bernard, Rachael D. Seidler

**Affiliations:** 1 Department of Psychology, University of Michigan, Ann Arbor, Michigan, United States of America; 2 School of Kinesiology, University of Michigan, Ann Arbor, Michigan, United States of America; University of Reading, United Kingdom

## Abstract

Older adults exhibit more bilateral motor cortical activity during unimanual task performance than young adults. Interestingly, a similar pattern is seen in young adults with reduced hand dominance. However, older adults report stronger hand dominance than young adults, making it unclear how handedness is manifested in the aging motor cortex. Here, we investigated age differences in the relationships between handedness, motor cortical organization, and interhemispheric communication speed. We hypothesized that relationships between these variables would differ for young and older adults, consistent with our recent proposal of an age-related shift in interhemispheric interactions. We mapped motor cortical representations of the right and left first dorsal interosseous muscles using transcranial magnetic stimulation (TMS) in young and older adults recruited to represent a broad range of the handedness spectrum. We also measured interhemispheric communication speed and bimanual coordination. We observed that more strongly handed older adults exhibited *more* ipsilateral motor activity in response to TMS; this effect was not present in young adults. Furthermore, we found opposing relationships between interhemispheric communication speed and bimanual performance in the two age groups. Thus, handedness manifests itself differently in the motor cortices of young and older adults and has interactive effects with age.

## Introduction

One of the most robust findings in the cognitive neuroscience of aging literature is the more diffuse, bilateral, task-related brain activity seen in older versus young adults during the performance of cognitive tasks [1]–[3]. A burgeoning literature investigating motor tasks reports a similar pattern of bilateral brain activation in older adults [4]–[7]. However, the literature is mixed regarding whether additional activity in the motor cortex during motor tasks is associated with better performance for older adults [4], [5], or has deleterious effects on behavior [8].

In order to better understand the pattern of bilateral activation in the motor cortex in older adults, it is important to take into account other factors that might be associated with bilateral motor cortical activity. One such factor is handedness; young adults who report low levels of hand dominance show more bilateral brain activation when performing unimanual actions [9], [10] and have more bilateral motor cortical representations [11]. Furthermore, young adults are more likely to self-report mixed use of their hands [12], [13], while older adults self-report more strongly lateralized behaviors, particularly towards the right hand [12]–[14]. Handedness is thus particularly interesting to investigate in older adults given that hand preference differs in young and older adults and this may influence motor cortical representations.

Greater bilateral brain activation is seen in left and less-strongly handed individuals [9], [10], [15]. This difference in bilateral activation may be related to the morphology of the corpus callosum. Both post-mortem and neuroimaging studies investigating callosal size have indicated that the corpus callosum is larger in volume in left-handed individuals [16], [17] or those that self-report less hand dominance [18] than in right- or strongly-handed individuals.

The corpus callosum is also susceptible to age-related structural changes. Age differences in both the size of the callosum [19], [20] and callosal white matter integrity have been noted [20]–[24]. More specifically, integrity of the anterior and midbody callosal tracts, which connect cortical regions involved in motor control and selective attention, exhibit reductions in older adults [22]. These callosal changes may underlie age differences in interhemispheric communication and interhemispheric inhibition (IHI), resulting in the bilateral brain activation patterns seen with age [8], [24], [25].

IHI is reduced in older adults and is related to increased bilateral brain activity [6], [25]. Because the degree of IHI is related to the structure of the corpus callosum [26]–[28], bilateral brain activation in older adults may be due to underlying differences in callosal structure and function. We have recently demonstrated that with age there are indeed shifts in the relationships between callosal structure and performance on bimanual tasks that rely on callosal integrity [20], [28]. In addition, there is an increase later in life in the incidence of mirror movements [30] and motor overflow [24], [30]. Though common in young children, motor overflow reduces during development [29], possibly due to myelination of the corpus callosum and increased IHI. With age, atrophy in the callosum is associated with differences in interhemispheric interactions and increasing motor overflow, resulting in bimanual interference during motor tasks [24].

We have previously demonstrated that less strongly handed young adults, defined as those with more equal manual dexterity for the two hands and reduced frequency of dominant hand use, show more ipsilateral MEPs [11]; these individuals also have larger corpus callosa [18]. However, older adults have smaller corpus callosa [19], [20], show more bilateral activity during the performance of motor tasks [4], [5], [7], and exhibit stronger hand dominance [12], [13] than young adults. What remains unknown is how handedness manifests itself in the aging brain in terms of motor cortical organization, interhemispheric communication, and bimanual performance, all of which are related to the corpus callosum to at least some degree.

Interhemispheric communication has classically been investigated with the Poffenberger paradigm, in which participants respond to lateralized visual stimuli with one hand or the other [31], [32]. The crossed (stimulus in the left visual field and right hand responses) minus the uncrossed reaction times results in a measure of interhemispheric transfer time known as the crossed-uncrossed difference, or CUD. The CUD is thought to rely at least in part on the corpus callosum [32], [33] because callosotomy patients have dramatically slower interhemispheric transfer times. Functional neuroimaging has also supported the notion that performance on the Poffenberger Paradigm relies on interhemispheric communication between the premotor cortices [34]. The CUD has been related to callosal size [35], [36] and microstructure using an electrophysiological measure of CUD [37]. Finally, the CUD has been shown to be longer in older adults [38] than young adults, and related to the strength of hand dominance in young individuals [11]. Given the known callosal changes with age, and the relationships between the corpus callosum and interhemispheric communication, the Poffenberger paradigm provides a useful behavioral metric of interhemispheric communication.

In the current study, we tested the hypothesis that hand dominance is differentially related to motor cortical representations in young and older adults. We used single pulse TMS to map motor cortical representations of the first dorsal interosseous (FDI) muscle in young and older adult participants recruited to represent a broad spectrum of handedness scores. We quantified the spatial extent of cortical representations as well as the number of contralateral and ipsilateral MEPs. We also quantified interhemispheric transfer time using the Poffenberger paradigm [31]. Finally, we used the bimanual and assembly components of the Purdue pegboard task [39] to quantify bimanual performance in order to further investigate the notion of shifts in brain structure-function relationships with age.

We predict that the relationship between handedness and ipsilateral motor cortical representations in young and older adults will be in opposite directions, in keeping with the hypothesis that there are qualitatively different structure-function relationships concerning the corpus callosum in these two age groups [23], [24], [40]. This would also support the hypothesis that handedness and age have interactive effects on the motor cortex. Specifically, we predict that in older adults, those with the largest number of ipsilateral MEPs would be those that are *more* strongly handed, which is the converse of what we recently reported for young adults [11]. We further hypothesize that interhemispheric transfer time will differentially relate to bimanual performance for young and older adults, analogous to what we reported in terms of relationships between callosal structure and bimanual coordination [20]. Our recent work indicates that over-activation and spreading of motor cortical representations in older adults have deleterious effects on motor performance [8], [41], thus leading us to hypothesize that we would see the same here.

## Methods

### Ethics Statement

All study procedures were reviewed and approved by the University of Michigan Institutional Review Board. Upon enrollment in the study, participants signed an approved consent form according to the principles of the Declaration of Helsinki.

### Participants

33 young adult (mean ± SD, 21 ± 2years, 8 male) and 23 older adult (70 ± 4, 9 males) participants were recruited from the University of Michigan and the surrounding community and were paid for their participation. Partial subsets of these data have been reported previously [11], [41]. Exclusion criteria included a history of neurological disease or damage, migraines, arthritis, head injury, psychiatric disorder, or recent changes in blood pressure medication (less than 6 months on the current drug and dosage). All participants were cognitively healthy as measured by the Mini-Mental State Exam [42] and the Mattis Dementia Rating Scale [43].

### Experimental Set-Up and Procedure

Testing occurred on two separate days. During session 1 participants completed the Edinburgh Handedness Inventory [44] to provide a self-report measure of handedness. The Tapping Circles and Tapping Squares from the Hand Dominance Test [45] were administered to provide graphomotor measures of handedness and laterality of dexterity for each hand. These tests required participants to tap dots in either small circles or small squares with a felt tipped pen using both the dominant and non-dominant hand (on separate trials). The number of circles and squares tapped in 30 seconds was recorded for each hand and used to calculate a laterality index. All participants completed both tapping measures. The order of hands and tests were counterbalanced across participants. Participants also performed the Purdue pegboard test [39] (right hand, left hand, bilateral, and assembly tasks, 30 seconds per trial, 3 trials per task). The Purdue pegboard was initially developed to assess the manual dexterity of potential assembly line workers [39] but is also used clinically for assessment of manual dexterity. In the bimanual condition, both hands were used simultaneously to put pegs into parallel rows of holes, and in the assembly condition the hands were used in an asynchronous fashion to build small spools using pegs, cylinders, and washers. The number of pegs (unimanual conditions), the number of pairs of pegs (bimanual condition), and the overall number of pieces (assembly condition) were recorded. Grip strength was measured using a hand dynamometer. Participants were asked to squeeze with maximal force. The performing hand was alternated trial to trial and three trials were completed for each hand. Laterality indices (LI) were calculated for the Edinburgh Handedness Inventory, Tapping Circles, Tapping Squares, unimanual Purdue pegboard, and grip strength as follows: (R-L)/(R+L), where R and L indicate right and left hand responses, respectively. Participants also provided health history information.

We used a computerized version of the Poffenberger paradigm [31] implemented with E-Prime 1.1 software (Psychology Software Tools, Inc). Participants made unimanual responses to white dots presented on a black screen to the left and right of a central fixation cross. Participants were instructed to respond as quickly as possible upon presentation of the white dot. The fixation cross was presented with a variable foreperiod (500, 650, 800, or 1000 ms) to discourage anticipatory responses. Responses were blocked by hand for 100 trials after which the response hand was switched. The ordering of left and right hand response blocks was counterbalanced across participants. 400 trials were completed per hand.

During the second session, participants underwent a TMS motor-mapping procedure. Participants sat comfortably in a chair with their head resting in a chin rest and their hands relaxed. A tight-fitting Lycra swim cap was placed on the head to allow for the marking of stimulation locations. Motor-evoked potentials (MEPs) were recorded from the FDI of both hands using 4 mm Ag/AgCl^−^ electrodes placed on the muscle in a belly-tendon arrangement. A ground electrode was placed on the lateral bone of the right wrist (ulna). MEP data were recorded and digitized at 2000 Hz using Biopac hardware and AcqKnowledge software (BIOPAC Systems Inc., Goleta, CA). Our system was carefully shielded by twisting the electrode wires, and all leads were secured in place so that they were not in contact with one another.

A Magstim 70 mm figure of eight coil with a Magstim Rapid stimulator (Magstim Company Ltd., Wales, UK) was used for TMS. The motor hot-spot for the FDI muscle for each hand was localized by stimulating at a high intensity. We first determined point Cz on every participant. From there, we measured 4 cm directly anterior and 4 cm lateral to point Cz. We then bisected the 90° angle made by drawing a line from the nasion to the inion and one between the two ears. A measuring tape was placed to connect the two 4 cm marks on the head, and the location where the measuring tape crossed our angle bisection line was used as our starting point. From there, the coil was placed on the head, 45° with respect to the midline, and in an anterior to posterior fashion. The front of the coil was oriented towards the front of the head. We began stimulating at a high intensity (approximately 70% of stimulator output) with the front of the coil lined up with the starting point previously described. Typically, this spot gave us quite strong motor activity using high levels of stimulation. However, in cases where we did not see any motor activity, we moved the coil in 1 cm increments, anterior, posterior, medial, and lateral, to the starting point. Resting motor threshold was then determined to the nearest two percent of stimulator output that elicited MEPs of at least 50 µVolts on three out of six consecutive stimulations while the target muscle was at rest (cf. [11], [41], [46], [47], [48], [49]). We then repeated the procedure of moving the coil in 1 cm increments around the hot-spot to ensure that we had found the correct spot and properly determined the motor threshold. If, when any of these locations were stimulated they showed MEP activity above 50 µVolts at a lower percentage of stimulation output, we continued to decrease the stimulator output at the new location, and repeated the procedure of stimulating the surrounding points as needed.

A 6×6 grid of points 12 mm apart was used for motor mapping in each hemisphere and the grid was designed to encompass the motor cortical hand representation. Grid location was determined based on anatomical landmarks such that the top of the grid was placed 2 cm infero-laterally from point Cz [50]. The grid extended 4.8 cm in the anterior direction and 1.2 cm in the posterior direction. Each site was stimulated 6 times at 110% of motor threshold with at least 6 seconds in between each stimulation trial. Stimulation was performed while the FDI was at rest, as determined with EMG monitoring during the experiment. Throughout the mapping procedure the coil orientation was maintained at a 45° angle with respect to the midline, while the coil was placed tangential to the head and held in an anterior to posterior orientation.

### Behavioral Data Processing

Reaction times from the Poffenberger paradigm were trimmed such that all responses faster than 100 ms (anticipatory responses) and greater than three standard deviations from the mean reaction time of an individual subject (attentional lapses) were omitted. Accuracy was measured as the proportion of button presses in response to the white dot, within the given timeframe. Any blocks where participants were below 66% response accuracy were also excluded from analysis. As a result, 6 young adults were excluded from analyses of the CUD, as they performed below 66% accuracy on the majority of the task blocks. Additionally, their average accuracy across blocks fell below 66%. The only instances of accuracies below 66% occurred in these participants. The accuracies ranged from 81.9% to 99.5% (mean = 91.7%) in the young adults (25.6% to 99.1% with a mean of 87.2% before the removal of these 6 participants), and from 91.9% to 99.2% (mean = 95.9%) in the older adults. There was a significant difference in the accuracy of the two groups (t_(50)_ = 3.08, p<.05). Given that our variable of interest, the CUD, is a difference score it corrects for subject-specific differences in reaction time, and is only computed using correct trials. In all cases, there were a sufficient number of trials to accurately compute the CUD (>600 trials). The CUD was computed by subtracting the mean uncrossed reaction time (trials where visual information was presented to the same hemisphere required to initiate the motor response) from the crossed reaction time (trials where visual information was presented to the opposite hemisphere required to make the motor response).

### MEP Data Processing

**Figure 1:**
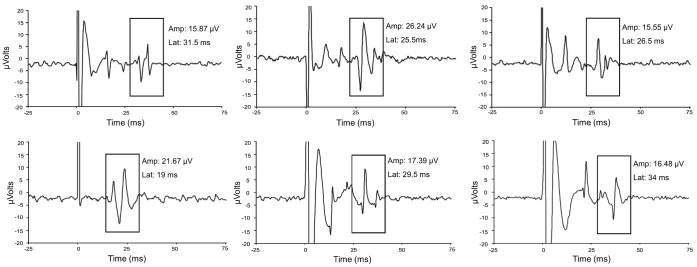
Ipsilateral MEP Traces. Example ipsilateral MEP traces in both young (top row) and older (bottom row) adults with an amplitude of approximately 15 µVolts. All traces have been time locked to show 25 ms of activity prior to the onset of stimulation, with stimulation occurring at time 0. The ipsilateral MEPs are highlighted using a rectangle to distinguish them from the stimulation artifact. The peak-to-peak amplitude and latency of each MEP is also presented.

EMG data were filtered online (10 and 500 Hz bandpass filtering) and digitized using a Biopac MP100 system with EMG 100C amplifiers and AcqKnowledge software. MEP onset latency was calculated as the point at which the MEP amplitude reached two standard deviations above the mean of the amplitude of the resting state muscle activity (oscillating around 0 µVolts) from 500 ms before the TMS pulse and 500 ms after any TMS-induced activity. The peak-to-peak amplitude of the resulting MEPs was also calculated. Both contralateral and ipsilateral MEPs were defined as those having a peak-to-peak amplitude of at least 15 µVolts [11], [41], comparable to the criterion used by Wasserman and colleagues [51]. This smaller cut-off was used in order to ensure that all ipsilateral activity was included. In our opinion, the use of a 50 µVolt cut-off results in the exclusion of a large number of real MEPs, and including them is crucial for a better understanding of motor cortical organization. The 15 µVolt cut-off was used for both contralateral and ipsilateral MEPs so as not to bias our estimates in the two cases. Example ipsilateral MEP traces are presented in Figure 1. Our recordings were characterized by very low noise, making even these smaller ipsilateral MEPs clearly visible. However, all ipsilateral MEPs were visually inspected to ensure that there was no muscle activity prior to the TMS and that the activity was not part of the stimulation artifact. The latency of the MEP was used to further confirm this. Even when using this low cut-off, the average amplitudes for the contralateral MEPs were (mean ± SD) 131.6 ± 71.6 µVolts and 176.1 ± 114.2 µVolts for the young and older adults, respectively. The average ipsilateral amplitudes were 40.0 ± 37.4 µVolts and 64.9 ± 99.4 µVolts for the young and older adults, respectively.

### Statistical Analyses

All of our statistical analyses were completed using R (www.rproject.org). Relationships between handedness and dexterity measures and CUD were assessed using Pearson product-moment correlation. The number of ipsilateral MEPs was investigated in both age groups in relation to handedness and dexterity measures. In these cases Poisson regression models were used. The Poisson distribution is used for evaluating count data where high frequency events are relatively rare [52], as we found to be the case with the occurrence of ipsilateral MEPs. Similar relationships with contralateral MEPs were investigated using Pearson product-moment correlation. Fisher’s r to z transform was used to investigate group differences in correlation coefficients. Additionally, we investigated relationships between average motor threshold and MEP amplitude (contralateral and ipsilateral) as well as the number of both contralateral and ipsilateral MEPs. The varying degrees of freedom reported for each test reflect the fact that not all individuals showed ipsilateral MEPs, and therefore were not included in analyses of amplitude. Similarly, several individuals were dropped from our analyses of CUD due to poor performance, but were included in analyses of laterality and the TMS measures.

## Results

### Handedness and Behavioral Measures

Young adults performed significantly better than older adults on the Mini-Mental State Exam (mean ± standard deviation; young adults = 29.79 ± .48, older adults = 28.87 ± 1.52; t_(54)_ = 3.26, p<.01) though both groups were approaching ceiling performance. There was a comparable nonsignificant trend on the Mattis Dementia Rating Scale indicating that young adults performed better than older adults (young adults = 142.88 ± .99, older adults = 142.26 ± 1.51; F_(54)_ = 1.85, p = .07). Table 1 shows the mean and standard deviation of the handedness and dexterity scores for both age groups. There were no significant differences between the two age groups on any of the handedness or laterality of dexterity measures (p>.05 in all cases), as expected given that we recruited across a wide range of handedness. However, the young adults performed better than the older adults on both the bimanual (larger number of pairs; t_(53)_ = −6.32, p<.001) and assembly (number of pieces assembled; t_(53)_ =  −7.76, p<.001) components of the Purdue pegboard task. Paralleling our prior work in young adults [11], we ran our further analyses of handedness and dexterity using our graphomotor performance measures (tapping circles and squares), and for parsimony, only tapping squares is presented. However, correlations between both tapping circles and tapping squares and our MEP measures are presented in Table 2. Correlations between the handedness and dexterity measures in the older adults are presented in Table S1.

**Table 1:** Mean (± SD) laterality indices for handedness assessments in older adults.

Handedness Assessments	Young Adults	Older Adults
**Edinburgh Handedness Inventory**	.25 (.59)	.53 (.56)
**Tapping circles**	.03 (.14)	.08 (.12)
**Tapping squares**	.03 (.09)	.07 (.09)
**Purdue pegboard**	.02 (.05)	.01 (.05)
**Grip strength**	.02 (.05)	.03 (.07)

**Table 2:** Correlations between the absolute value of the Tapping Squares and Circles laterality indices and the number of contralateral and ipsilateral MEPs (assessed using Poisson regression models) in young and older adults.

	Tapping Squares		Tapping Circles	
	YA	OA	YA	OA
**Contralateral MEPs**	r_(31)_ = .02, p>.8	r_(21)_ = .72, p<.001	r_(31)_<.01, p>.5	r_(21)_ = .11, p>.6
**Ipsilateral MEPs**	chi-squared = 3.98, p<.05	chi-squared = 8.42, p<.01	chi-squared = 5.65, p<.05	chi-squared = .008, p>.9

**Figure 2:**
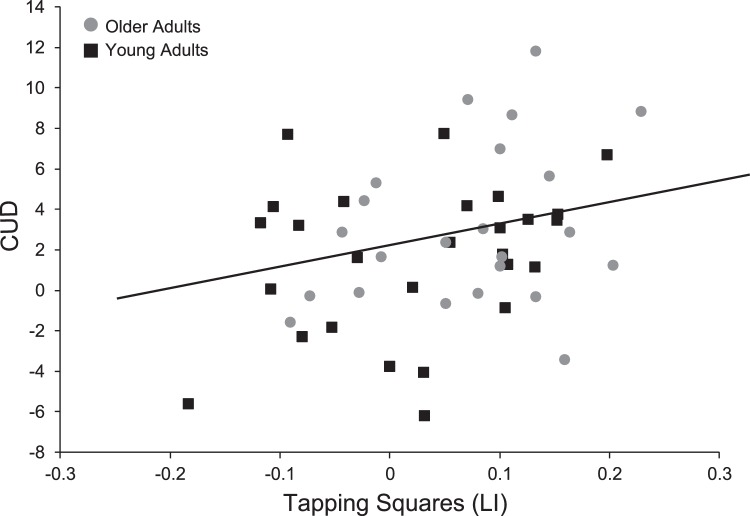
Relationships Between CUD and Handedness. CUD plotted as a function of handedness measured with the Tapping Squares LI in both the young (black) and older (grey) adults. There is a significant correlation when the two age groups are combined (r_(48)_ = .32, p<.05).

The mean CUD time calculated from the Poffenberger paradigm for the young adults was 1.6 ± 3.7 msec and for the older adults it was 3.1 ± 3.9 msec. There was no significant difference between the two age groups (t_(48)_ = 1.37, p>.1). The mean reaction time for each hand and visual field are presented for the young and older adults in Table 3. Correlations between CUD and laterality measures for the young adults were reported previously [11]. In summary, we found that less strongly handed individuals have CUD times closest to zero, with left-handed individuals exhibiting the fastest, and often negative, CUD times. The correlation between CUD and tapping squares in older adults was not significant (r_(21)_ = .30, p = .17). Thus, degree of handedness does not predict interhemispheric transfer time in older adults. However, when we pooled both age groups we found that there was a significant correlation between the tapping squares measure of handedness and CUD (r_(48)_ = .32, p<.05; Figure 2). Left-lateralized and less strongly lateralized individuals had the fastest, and often negative, CUD times.

**Table 3:** Mean (±SD) reaction time for each hand and visual field in the Poffenberger paradigm for young and older adults.

	Left Visual Field		Right Visual Field	
	YA	OA	YA	OA
**Left Hand**	241.2 (54.3)	288.6 (57.5)	250.5 (42.5)	299.0 (53.7)
**Right Hand**	241.3 (47.7)	290.3 (54.4)	247.7 (40.8)	290.6 (42.9)

**Figure 3:**
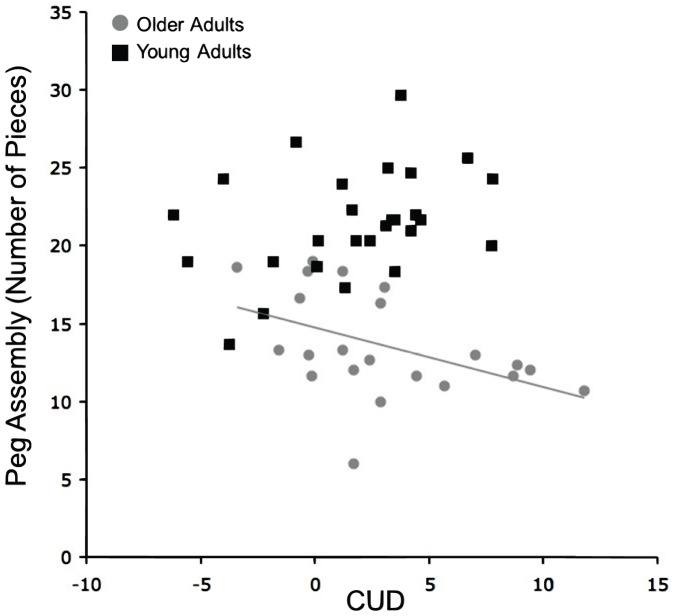
Relationship Between CUD and Bimanual Performance. Correlation between performance on the assembly component of the Purdue pegboard and CUD for the older adults (gray) and the young adults (black). There is a significant correlation in the older adults (r_(21)_ =  −.45, p<.05), though the correlation is not significant in the young adults (r_(25)_ = .36, p = .07). The strength of these correlations are significantly different (z = −2.67, p<.01).

Additionally, we examined whether CUD predicted performance on the bimanual or assembly tasks of the Purdue pegboard. We found a significant negative correlation between CUD time and performance on the assembly component in older adults (r_(21)_ = −.45, p<.05) indicating that those with CUDs closest to zero, or negative, assembled more pieces on the pegboard. This supports the notion that faster interhemispheric interactions contribute to successful bimanual task performance. Young adults exhibited a positive relationship although it was not significant (r_(25)_ = .36, p = .07; Figure 3). The strength of these correlations significantly differed between the two age groups (z = −2.67, p<.01).

### TMS Metrics and Handedness

There were no significant correlations between motor threshold and the number of contralateral (r_(51)_ = .05, p>.7) and ipsilateral MEPs (Poisson regression model; chi-squared = 2.13, p>.1), nor the ipsilateral MEP amplitude (r_(43)_ =  −.03, p>.8), though there was a significant correlation with the contralateral MEP amplitude (r_(52)_ =  −.31, p<.05). However, the direction of the relationship between contralateral amplitude and motor threshold was in the direction opposite to what we would hypothesize if motor threshold were influencing the MEPs. The lack of correlation between motor threshold and the other MEP measures indicates that our results are not being driven by motor threshold, and that they stand above and beyond any potential confounding influence of motor threshold.

Similar to our previous work [41], group differences were seen between the young and older adults in general TMS metrics (motor threshold and latency). Please see Table S2 for these values by group. However, because our analytical approach is focused here on individual differences, we report these group level findings as supplementary materials.

**Figure 4:**
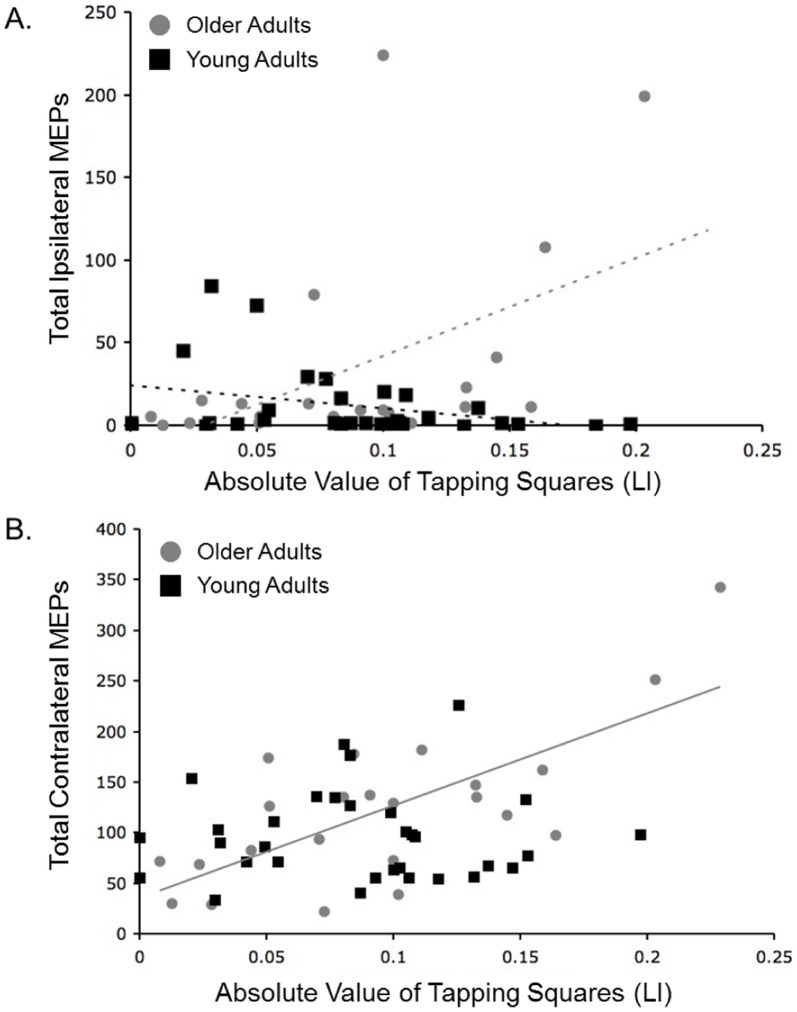
A. Ipsilateral MEPs and Handedness. Total number of ipsilateral MEPs plotted as a function of degree of handedness as measured by the absolute value of the tapping squares measure for the older (gray) and young (black) adults. The Poisson regression models indicate statistically significant relationships for both age groups (older adults: chi-squared = 8.42, p<.01; young adults: chi-squared = 3.98, p<.05) though they are in opposite directions. Overlaid lines serve to highlight and differentiate the relationships. **B. Contralateral MEPs and Handedness.** Total number of contralateral MEPs plotted as a function of degree of handedness, as measured by the absolute value of the tapping squares measure for the older (gray) and young (black) adults. The Poisson regression models indicate a statistically significant relationship in the older adults (r_(21)_ = .72, p<.001), though there is no relationship in the young adults (r_(31)_ = .02, p>.8).

Ipsilateral MEPs were seen in all but 5 participants (4 young adults and 1 older adult). Poisson regression models indicated that young adults showed a significant negative relationship between laterality of dexterity as measured by the absolute value of the tapping squares measure and the number of ipsilateral MEPs (degree of handedness; chi-squared = 3.98, p<.05). In contrast, older adults showed a significant positive relationship (chi-squared = 8.42, p<.01; Figure 4a; Table 2). That is, more strongly lateralized older adults showed a *greater* number of ipsilateral MEPs while more strongly lateralized young adults exhibited *fewer*. In contrast, there was no relationship between the number of ipsilateral MEPs and laterality of dexterity measured using the absolute value of the tapping circles measure, though we previously reported such a relationship in young adults (Table 2; Bernard et al., 2011). We also found a significant positive relationship between the number of contralateral MEPs and the absolute value of the tapping square measure of laterality in the older adults (r_(21)_ = .72, p<.001; Figure 4b) while there was no relationship in the young adults (r_(31)_ = .02, p>.8). The strength of these two relationships differed significantly between the two groups (z = 3.05, p<.01). More lateralized older adults had a larger number of contralateral MEPs, comparable to the pattern seen with the ipsilateral MEPs.

### Symmetry of MEP Distributions and Interhemispheric Transfer Time

**Figure 5:**
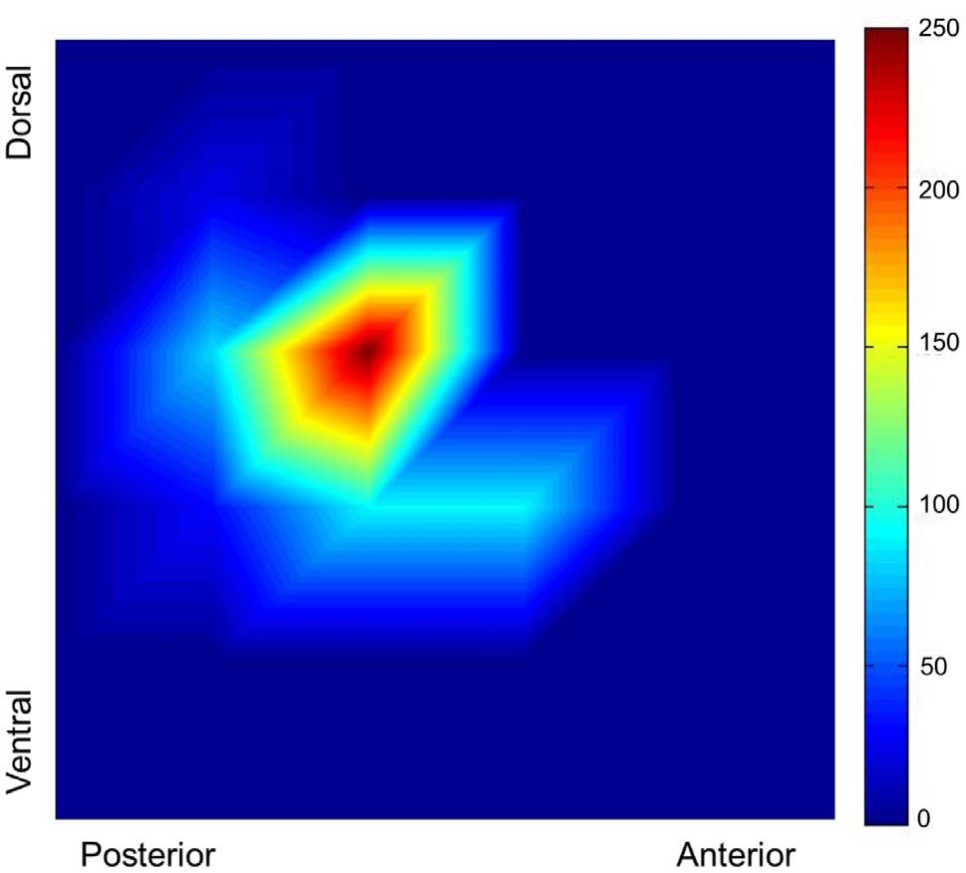
Representative MEP Distribution. The MEP distribution of the dominant (left) hemisphere of a right-handed young adult is presented to illustrate the created distributions. The color bar indicates the average amplitude at each point (in µVolts).

**Figure 6:**
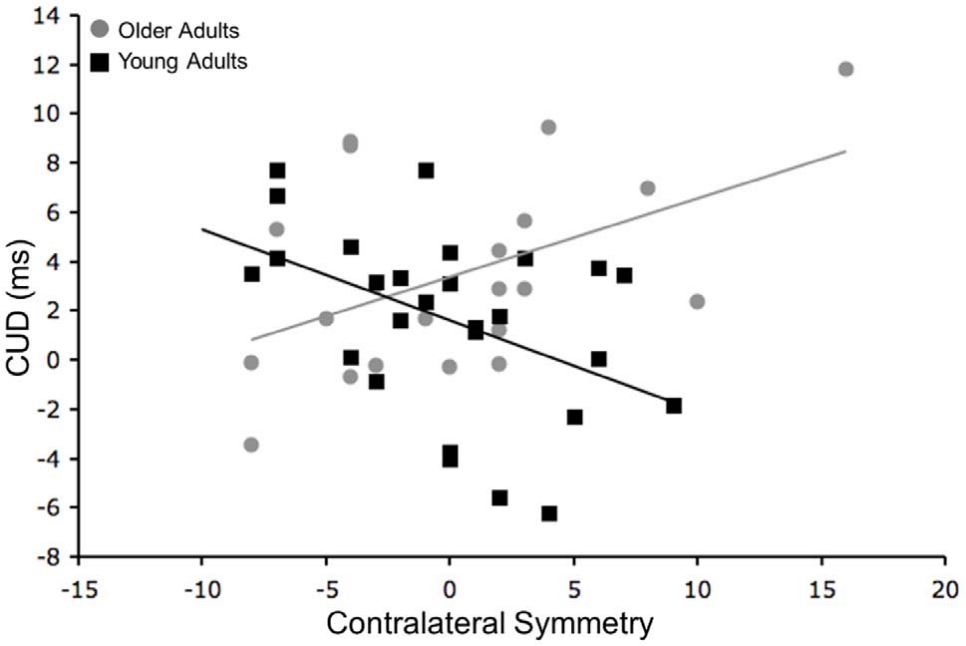
CUD and MEP Distribution Symmetry. Correlation between CUD and contralateral MEP distribution symmetry (# points dominant - # points non-dominant) for the older adults (gray) and the young adults (black). The correlations in both age groups are significant (young adults: r = −.53, p<.01; older adults: r = .49, p<.05), and the strength of these correlations is also significantly different (z = −3.72, p<.001).

Our TMS procedure allowed us to create plots of the distributions of the sites that elicited MEPs in both the dominant and non-dominant hemispheres. These distributions are referenced to point Cz, and represent the 36 points used for stimulation. The average amplitude at each stimulation point was calculated. We then plotted these averages across a grid to create a 2D plot of the distribution of the MEPs. An example distribution from one representative participant is presented in Figure 5. The symmetry of the distributions was calculated by subtracting the number of points in the non-dominant representation with a mean MEP amplitude of at least 15 µVolts from those in the dominant representation with a mean MEP amplitude of at least 15 µVolts, using the contralateral distributions only. While there was no age difference in symmetry (F_(1,53)_ = .93, p>.3), symmetry was differentially correlated with CUD in the young and older adults. That is, we found significant correlations in both age groups but of opposite signs (young adults: r_(25)_ =  −.53, p<.01; older adults: r_(19)_ = .49, p<.05; Figure 6), with a significant difference between the strength of these two correlations (z = −3.72, p<.001). The positive correlation in the older adults indicates that those with larger non-dominant representations had faster CUD times. The shift in the pattern of the relationships between the two age groups parallels what we found for CUD and Purdue pegboard performance as described above.

## Discussion

We investigated whether there are differences with age in the relationships between handedness, laterality of dexterity and motor cortical organization. We provide evidence that handedness manifests itself differently in terms of both neurophysiology and behavior in young versus older adults. Our results support the notion of an age-related shift in the relationships between handedness, motor cortical organization, and interhemispheric interactions. Our key findings are as follows: 1) older adults with more strongly lateralized hand preference exhibit more ipsilateral MEPs, while in young adults ipsilateral MEPs are more common in less strongly lateralized individuals, and 2) the relationship between CUD and bimanual task performance indicates that the conditions for optimal performance in older adults differ from those in young adults. This may be due to qualitative age differences in interhemispheric interactions, perhaps related to handedness. We also provide evidence indicating that the more bilateral motor cortical representations in older adults may be compensatory; that is, older adults with more symmetrical MEP distributions had faster CUD times.

### Age, Handedness, and Neurophysiology

We have previously reported that in young adults, less strongly lateralized individuals show a larger number of ipsilateral MEPs [11]. Here we demonstrate that in older adults, ipsilateral MEPs are more common in those that are *more* strongly lateralized in terms of manual dexterity. Similarly, more strongly handed older adults show more contralateral MEPs, while there is no relationship between handedness and contralateral MEPs in young adults. In both of these instances, there is likely at least some degree of callosal involvement. Furthermore, we know that there are age differences in callosal structure [19]–[23] (both overall size and also microstructure). We provide support for an age-related shift in the structure-function relationship of the corpus callosum [28], [40], though handedness likely interacts with these effects.

However, it is notable that in the young adults relationships are seen with both the tapping circles and squares measure of laterality. In the older adults, we only see relationships with the tapping squares measure. This somewhat limits our ability to generalize about these findings, though we have demonstrated that laterality measured using tapping squares is manifest differently in the motor cortex of older adults. It is unclear why we do not see a comparable pattern with the tapping circles measure in the older adults. One possibility is task difficulty. The circles are much smaller than the squares and this may have made the task more difficult for the older adults, resulting in a less accurate measure of laterality. Regardless, this lack of a relationship was somewhat surprising and future work is needed to further investigate this difference with respect to the findings in the young adults.

We speculate that age differences in ipsilateral MEPs may be a result of differences in the corpus callosum. Handedness may have differential effects on the corpus callosum with age. Prior work has demonstrated that less strongly handed and left-handed individuals have larger corpus callosa [16]–[18], [53]. In young adults, less strongly lateralized individuals show more ipsilateral MEPs, perhaps due to decreased interhemispheric inhibition (IHI). However, in older adults, the pattern is reversed such that more strongly lateralized older adults show a greater number of ipsilateral MEPs. Callosal connections between the primary motor cortices are known to be primarily inhibitory in nature. Furthermore, callosal microstructure is correlated with inhibition of the contralateral primary motor cortex, as measured using the ipsilateral silent period [28], though the direction of this relationship shifts in older adults [24]. Given this known inhibitory relationship between the two motor cortices, while stimulating over one hemisphere, we would speculate that there is inhibition of the other hemisphere, mediated by the corpus callosum. Because there are known differences in callosal microstructure with age and a known decrease in IHI with a concomitant increase in interhemispheric facilitation [24], we suggest that this is a potential mechanism resulting in the ipsilateral MEPs seen in our study. Our findings are consistent with this notion, in that the most strongly lateralized individuals, some of whom are right-handed, show greater ipsilateral MEP activity. However, handedness may interact with these processes resulting in differential aging effects across the handedness spectrum. That said, we did not directly measure IHI in our participants, thus this remains speculative. Future research would benefit from including measures of IHI and corpus callosum structure in investigations of handedness.

Though we propose that the increase in ipsilateral MEPs in older adults may be due to changes in the corpus callosum and interactions with handedness, it is important to note that the pathways underlying these MEPs are unclear. While the majority of research using TMS has suggested that ipsilateral MEPs are due to ipsilateral branching of the corticospinal tracts [54], [55], our previous work showing relationships between ipsilateral MEPs and interhemispheric communication point to a potential callosal mechanism [11]. Furthermore, it is thought that the majority of the ipsilateral corticospinal tracts project to axial muscles [56] making it unlikely that these tracts are underlying our reported ipsilateral MEPs. Indeed, recent evidence in non-human primates indicates that any ipsilateral inputs to the limbs are weak and indirect [57], making this an unlikely mechanism. However, future research would benefit from a direct investigation of the mechanisms resulting in ipsilateral MEPs in both young and older adults.

Finally, given the known age differences in callosal microstructure [22]–[24], [28], and the possibility that the balance of inhibitory and excitatory connections via the corpus callosum differ by age [24], [40], it seems plausible that the increase in ipsilateral MEP activity seen with age is due, at least in part, to these callosal effects. Across the handedness spectrum there is variance in the degree of interhemispheric communication, which is reliant upon the corpus callosum. Handedness may interact with age-related differences in callosal microstructure, resulting in differing relationships between ipsilateral MEP activity and handedness in young and older adults. Future investigations are however needed to test this notion.

### Age, Handedness, and Behavior

We did not observe a significant age difference in interhemispheric transfer time as measured by the CUD. In contrast, Jeeves & Moes [38] found that older adults show slower CUD times. It is unclear why we did not see this difference. Though our results trend towards older adults having slower CUDs, the difference was not significant. This may be due, at least in part, to the inclusion of participants across the handedness spectrum. We have shown previously that degree of handedness in young adults was correlated with CUD time [11], and a similar pattern is seen here when pooling across the two age groups. Including individuals with a wide range of handedness scores may have diluted any age differences.

However, it is notable that several young adults but no older adults were dropped from our analysis of the Poffenberger paradigm data due to poor performance. Given the generally high accuracies in both age groups (with the exception of the omitted participants), this is likely a result of motivation, rather than task difficulty or experimental design. Indeed, if task difficulty or design were an issue we may expect an equivalent or greater number of older adults would have had difficulty performing the task. Rather, it is likely that our young adults disengaged from the task, despite that we provided them with several rest breaks and reminders to stay focused on the task.

Our findings of a differential relationship between CUD and bimanual coordination based on the assembly component of the Purdue pegboard may be due to interactions between handedness and callosal neurophysiology in aging. In older adults, those with faster CUD times had better performance on the assembly task. Generally, those individuals are less strongly handed, and in the older adults may have higher levels of IHI, based on our ipsilateral MEP findings. Individuals with higher levels of IHI are able to maintain proper inhibition between the two hemispheres, which is important for asynchronous bimanual tasks [58]. Furthermore, it may be that less strongly lateralized older adults with less change in callosal microstructure show greater IHI, also contributing to bimanual performance. This may be the case for the older adults with the fastest CUD times. Thus, the effects of handedness on neurophysiology in aging also have behavioral relevance.

### Compensation and Motor Representations

We were able to investigate indirectly whether the bilateral task-related functional activation seen in the motor cortex in older adults plays a compensatory role, or if it has deleterious effects by examining the symmetry of the motor cortical representations and its relationship with interhemispheric transfer time. Contrary to our initial predictions, we found that older adults with larger non-dominant representations than dominant have faster interhemispheric transfer time. Saron and colleagues [59] have shown that those with more bilateral signal processing demonstrate shorter CUD times. Similarly, when performing complex letter matching tasks, older adults show an advantage in cases where relevant information is presented to both visual hemifields [60]. Spillover between the two hemispheres may aid in this processing. In older adults, the larger non-dominant representation may allow for bilateral signal processing and provide a compensatory mechanism in tasks where engagement of both hemispheres is advantageous.

To this point, the evidence for compensatory brain activation in the motor cortex is relatively mixed [4], [5], [8], [41]. Though we previously provided evidence in support of dedifferentiation with more motor activation or larger representations associated with *poorer* performance in older adults [8], [41], the tasks used in those studies were different from the measure of CUD described here. Our prior work showed that longer reaction times were associated with more bilateral motor cortical activity [8] and more expansive motor cortical representations [41]. Reaction time tasks may rely more on inhibitory circuits to prevent spillover, which is detrimental to performance. Thus, whether or not the bilateral task-related activation seen during motor processes in older adults is compensatory or a result of dedifferentiation may be task-dependent.

Finally, it is worth noting that we used a cross-sectional design in this study, and our behavioral and neurophysiological data were collected on separate days. Because we assessed two age groups we are unable to make any inferences about changes with age. However, we do note several interesting differences between our young and older adults with respect to relationships between handedness and motor cortical representations. Our results indicate that the relationships between both contralateral and ipsilateral MEPs and handedness are in the opposite direction in young and older adults. Because we used a cross-sectional design, we were unable to investigate whether this is a difference due to changes across the lifespan, or due to a cohort effect based on our sample. However, follow-up longitudinal studies investigating the interaction between handedness and neurophysiology would be an interesting next step.

Motor cortical excitability is known to be different at rest as compared to during task performance [61], and may vary on a day-to-day basis based on a variety of factors such as fatigue or time of day. It is important to consider these issues in light of our correlations between motor cortical symmetry measured at rest and task performance on the Poffenberger paradigm. However, because of this, one might expect that finding a significant correlation would be *less* likely, though we report robust correlations. Based on our data, resting representations may be in some way related to motor cortex function during task performance, regardless of the potential differences in motor cortical excitability that may occur due to our design. Further research would however be needed to investigate this.

### Conclusions

We provide evidence for differential manifestation of handedness in both brain and behavior in older adults. More strongly lateralized older adults show a greater amount of ipsilateral MEPs, while in young adults more ipsilateral MEP activity is seen in less strongly lateralized individuals. This provides support for the notion that there is a fundamental shift in the structure-function relationship of the sensorimotor regions of the corpus callosum of older adults. Furthermore, the differing neurophysiology of handedness in older adults may impact bimanual performance. Perhaps then, callosal changes with age vary across the handedness spectrum resulting in differences in interhemispheric interactions amongst these older individuals.

## Supporting Information

Table S1 **Correlation coefficients between handedness measures in older adults.** Significance of correlations is indicated (#p<.1, *p<.05, **p<.01, ***p<.001).
(DOC)

Table S2 **Mean (± SD) motor threshold, contralateral, and ispsilateral MEP latencies by age group and hemisphere of stimulation.** Significant main effects of age are indicated (**p<.01).
(DOC)
