# Supplementary material for: Hand Dominance and Age Have Interactive Effects on Motor Cortical Representations
Source: PLoS One. 2012 Sep 25;7(9):e45443. doi: 10.1371/journal.pone.0045443 (PMC3458089; doi:10.1371/journal.pone.0045443)
Supplement: Table S1 — Correlation coefficients between handedness measures in older adults. Significance of correlations is indicated (#p<.1, *p<.05, **p<.01, ***p<.001). (DOC) [file pone.0045443.s001.doc]

**Table S1. Correlation coefficients between handedness measures in older adults.**

|  | Edinburgh | Squares | Circles | Peg | Grip |
| --- | --- | --- | --- | --- | --- |
| Edinburgh | 1 | .59** | .80*** | .27 | .58** |
| Squares | - | 1 | .73*** | .38^#^ | .23 |
| Circles | - | - | 1 | .30 | .33 |
| Peg | - | - | - | 1 | .09 |
| Grip | - | - | - | - | 1 |

**Significance of correlations is indicated (#p<.1, *p<.05, **p<.01, ***p<.001).**

**Table S2. Mean (+**SD) motor threshold, contralateral, and ispsilateral MEP latencies by age group and hemisphere of stimulation.

|  | Dominant Hemisphere | | Non-Dominant Hemisphere | |
| --- | --- | --- | --- | --- |
|  | YA | OA | YA | OA |
| Motor Threshold (% Stimulator Output)** | 58.51 (4.94) | 64.5 (11.06) | 57.75 (5.69) | 64.3 (9.15) |
| Contralateral MEP Latency (msec)** | 20.44 (3.62) | 24.37 (3.80) | 20.29 (3.82) | 22.87 (4.16) |
| Ipsilateral MEP Latency (msec) | 25.77 (9.90) | 25.39 (7.63) | 20.43 (12.90) | 24.84 (7.40) |

**Significant main effects of age are indicated (**p<.01).**
